# Influence of Hydroxyethylmethyl Cellulose Admixture on the Hydration Process and Mechanical Properties of Modified Gypsum Composites

**DOI:** 10.3390/ma19040652

**Published:** 2026-02-08

**Authors:** Iwona Wilińska, Karol Prałat, Małgorzata Brych-Dobrowolska

**Affiliations:** Faculty of Civil Engineering, Mechanics and Petrochemistry, Warsaw University of Technology, Łukasiewicza 17, 09-400 Płock, Poland; karol.pralat@pw.edu.pl (K.P.); malgorzata.dobrowolska@pw.edu.pl (M.B.-D.)

**Keywords:** gypsum composites, hydration process, hydroxyethylmethyl cellulose

## Abstract

Gypsum is one of the main binding materials used in the construction industry. Its properties can be modified by the addition of chemical admixtures that may influence the hydration process and the microstructure of the hardened material. An important group of such admixtures comprises cellulose ethers. The aim of this study was to conduct an in-depth analysis of the effects of hydroxyethyl methylcellulose (HEMC) on the hydration and mechanical properties of gypsum. HEMC was applied in various amounts (ranging from 0.5 to 7% by mass of gypsum); the water-to-gypsum ratio was 0.75. The hydration process was investigated using calorimetry, thermal analysis, and infrared spectroscopy. Compressive and bending strength tests were also performed. The results of calorimetric measurements show that the presence of HEMC led to delayed hydration and prolonged gypsum crystallization, particularly at higher admixture dosages. No formation of new phases in the gypsum paste was observed in the presence of HEMC. However, the admixture modified the microstructure of the hardened material, as reflected by increased compressive and bending strength. This effect is most likely associated with the slower precipitation of gypsum crystals in the presence of HEMC.

## 1. Introduction

Cement, gypsum, and lime are important binding materials widely used in the construction industry. Concrete, which is composed of three main components—cement, aggregate, and water—is the most commonly used building material in the world. As a result, there is a significant global demand for cement [1].

Cement was invented in the nineteenth century when a method for producing a binder from a mixture of calcined limestone and clay was patented. Portland cement consists of Portland clinker—composed primarily of calcium silicates (alite and belite), tricalcium aluminate, and the ferrite phase—and ground gypsum. It is a hydraulic binder, meaning that hardening occurs both in water and in air in the presence of water. The setting and hardening of cement are based on reactions between its components. In general, the nature of the solid products formed depends on the hydration conditions and the proportions of the reactants. The hydration of alite and belite leads to the formation of the hydrated calcium silicates with variable chemical composition and usually a low degree of crystallinity (C-S-H phase), as well as calcium hydroxide. During the initial stage of hydration, gypsum reacts with aluminates to form ettringite, which is subsequently converted into monosulfate [2]. Gypsum is present in cement in amounts of several percent; however, it is also widely used as an independent binder.

Unlike Portland cement, which was developed relatively recently, gypsum plaster is among the oldest building materials known [3,4]. It was already used in ancient times [2]. Gypsum belongs to the class of air hardening binders. It should be noted that although the commercially available material is commonly referred to as “gypsum”, it is chemically calcium sulfate hemihydrate (CaSO_4_·0.5H_2_O), that is, bassanite. Gypsum in the strict chemical sense—calcium sulfate dihydrate (CaSO_4_·2H_2_O)—forms upon the contact of hemihydrate with water during the hydration and hardening process. On the other hand, natural gypsum (gypsum rock) is the raw material used for the production of commercial gypsum through thermal and mechanical processing [3]. The setting process of gypsum is based on the hydration of the hemihydrate, which results in the formation of calcium sulfate dihydrate (CaSO_4_·2H_2_O).

Because the chemical and phase composition of a gypsum binder is significantly simpler than that of Portland cement, the mechanism of its hydration process is also simplified. However, various factors influence the setting, hardening, and microstructure formation of gypsum and, consequently, the properties of the binder. This means that the mechanism of gypsum hydration, although it has been studied for many years and is already quite well understood, still arouses interest among researchers.

Depending on specific requirements, various types of chemical admixtures can be introduced into the gypsum binder, similarly to cement, in order to modify its properties [4]. Among the admixtures used in gypsum materials, an important group is polymer compounds, represented by cellulose ethers [3]. This group of admixtures has the ability to retain water and thus prevent uncontrolled water loss through the porous material [3,5,6]. The mechanism of water retention can be twofold, depending on the polymer concentration: through water absorption and through the formation of three-dimensional hydrocolloidal polymer networks [6]. Czaderna et al. [3] investigated gypsum containing different cellulose derivatives, namely hydroxyethyl methylcellulose (HEMC), hydroxypropyl methylcellulose (HPMC), and hydroxypropyl cellulose (HPC). They found that these polymers influence the setting time and significantly modify the morphology of the final hardened material. The setting process of gypsum containing polymers is longer, especially when HPMC is used, compared to gypsum without chemical admixtures. The authors explained this effect by the presence of hydrophilic groups in the additives, which strongly bind water molecules. This hinders water binding in the gypsum crystals [3]. The authors of [7] concluded that HPMC delays the setting and hardening of the gypsum binder; consequently, the induction period is longer, the hydration rate is slower, and the crystal nucleation rate is lower. The retarding effect of cellulose ether admixtures, such as HEMC, was also observed in [5]. On the other hand, Zhang et al. [8] found that HPMC slightly accelerates the early hydration of gypsum by promoting the nucleation step. Other organic admixtures tested in that study [8], i.e., polycarboxylate (PC) and citric acid (CA), exhibited a retarding effect. The study reported in [9] showed that the HEMC admixture can either accelerate or retard the setting of gypsum, depending on the polymer properties (viscosity and molecular weight) and the water-to-gypsum ratio. Liu et al. [10] found that hydroxypropyl starch (HPS) is adsorbed onto binder particles through complexation and, depending on the dosage, can act as either an accelerator or a retarder. In contrast, HPMC exhibited a setting-accelerating and structure-thickening effect across the entire dosage range used in [10]. One of the important mechanisms of interaction of polysaccharides in the hydrating gypsum system is adsorption, which can occur through complexation. This process depends, among other factors, on the molecular weight of the polymer [10].

In our research on the impact of polymers on the properties of gypsum, HEMC was selected as a representative of this group of admixtures. It is one of the most commonly applied admixtures for modifying the properties of gypsum materials, and its influence on macroscopic effects such as delayed setting and modified rheology is quite well documented. However, its interaction with calcium sulfate hemihydrate depends on multiple factors, including the polymer properties, dosage, and its complex influence on the kinetics and mechanisms of hydration. In our previous research, we observed that an increase in the amount of polymer in the sample led to an extension of the hydration time, as recorded during calorimetric measurements performed using both isothermal and diathermy methods. Depending on the amount of polymer used, the hydration time increased from several to several dozen minutes [11,12].

The addition of 1% HEMC polymer increases the hydration time. Moreover, the most significant change is observed up to 1.0% HEMC. A delay in reaching maximum hydration can also extend the crystallization time and thus influence the microstructure. During the hydration and crystallization of hemihydrate, changes in total porosity and pore structure are observed depending on the initial water-to-gypsum ratio. During aging, excess noncrystalline water evaporates from the pores, leading to a decrease in the density and thermal conductivity of the samples [13].

Summarizing, polymers such as HEMC can significantly affect the setting time and hydration of gypsum, as well as alter the crystallization mechanism. This has both advantages (e.g., workability modification adjusted to the intended use of a gypsum mixture) and disadvantages (often delayed strength development). Changing the hydration time may lead to problems with the uniform hardening of gypsum and disrupt its nucleation by modifying the crystal growth mechanism. Furthermore, depending on the amount of HEMC and its molecular weight, extremely viscous pastes can be obtained [5], which may influence the mechanism of action of the admixture in the system. The literature review shows that, although some generalized conclusions can be drawn, the effect of polysaccharide admixtures on gypsum properties depends not only on the type of chemical admixture but also on its dosage.

It follows from the above that continuous research on modified gypsum is necessary, as changes in setting time depend on the type of polymer, its purity, degree of substitution, and environmental conditions. Owing to extensive research and development on the use of polymers as admixtures in gypsum, it is possible to select the appropriate type and amount of polymer to achieve a compromise between setting time, strength, and ease of application. Research on polymer-modified gypsum is important because it enables the deliberate shaping and optimization of material properties, adapting the material to specific applications. This creates new opportunities for the broader use of gypsum materials.

To sum up the above, it can be stated that various factors influence the morphology of CaSO_4_·2H_2_O grains formed during the hydration of hemihydrate, which in turn affects the properties of the hardened binder. Despite the substantial amount of research on the mechanism of gypsum hydration, there remains a need for further study of this topic. This particularly applies to the physicochemical processes occurring in hardening gypsum under the influence of chemical admixtures. The effect of chemical admixtures on the hydration of gypsum binders has not yet been fully investigated. In this work, we further investigated in greater detail the influence of HEMC on the physicochemical hydration processes of gypsum binders and their properties.

The primary aim of the study was an in-depth analysis of the influence of HEMC on the hydration process of gypsum and on the mechanical properties of gypsum composites. In the experiment, the polymer was used as an admixture in weight ranges from 0.5 to 7% relative to pure gypsum. The following methods were used to study the effect of the admixture on the hydration process: calorimetry, thermal analysis, and infrared spectroscopy. Understanding the influence of polymers such as HEMC on gypsum hydration can provide valuable insights that may also be relevant to broader areas of materials science and construction technology. Some of the results were compared with those obtained for Portland cement.

## 2. Materials and Methods

**Figure 1:**
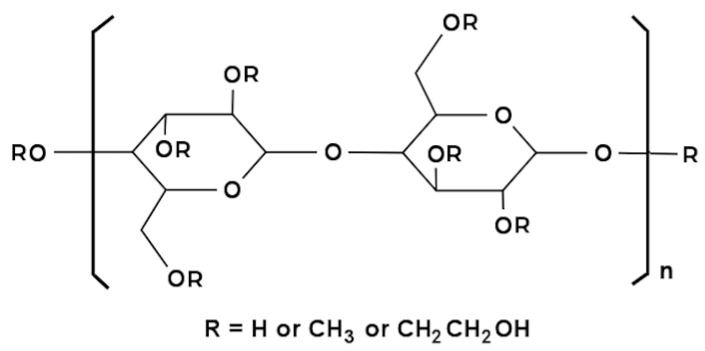
Chemical formula of HEMC.

The main materials used in this work were: commercially available gypsum powder (90–99 wt.% of bassanite, hemihydrate CaSO_4_∙0.5H_2_O) (G), with properties consistent with the safety data sheet [14], and Methyl 2-hydroxyethyl cellulose (HEMC) [15] with the chemical formula shown in Figure 1. For comparison, commercially available Portland cement CEM I 42.5 N (PC) with the following oxide composition was also used: SiO_2_ 19.4%, Al_2_O_3_ 4.88%, Fe_2_O_3_ 2.96%, CaO 63.4%, SO_3_ 2.71%, MgO 1.74%, Na_2_O + K_2_O 0.96%.

### 2.1. Gypsum

The material used in the research was building gypsum (Dolina Nidy, Pinczow, Poland). This gypsum is applied in construction, renovation, and finishing works inside buildings, as well as for producing mortars and prefabricated elements. A gray-yellow gypsum powder, widely available on the market and compliant with standard requirements, was selected. Some physical properties of the gypsum powder used in the study can be found in [16] and in the safety data sheet [14].

### 2.2. Polymer

The polymer used in this research was produced by Sigma-Aldrich (Poznań, Poland). Its molar mass was 858.9 g/mol. HEMC is a white, non-toxic powder that dissolves well in both hot and cold water and does not precipitate when heated. The viscosity of a 2% solution at 20 °C ranges from 15,000 to 20,500 mPa·s [15].

### 2.3. Sample Preparation

In the case of gypsum pastes, commercially available gypsum (calcium sulfate hemihydrate) was mixed with distilled water at a water-to-gypsum ratio of 0.75. The amounts of HEMC used were 0, 0.5, 3, and 7% of the dry gypsum weight. The adopted dosages of HEMC enabled the investigation of its effect on the hydration and mechanical properties of gypsum both at typical polymer dosages and at increased contents.

After combining the dry and wet components, the samples were mixed by hand for 1 min and then poured into molds. For calorimetric tests, the slurries were introduced into the calorimeter 1 min after the dry ingredients were combined with water. The measurement time was recorded from the moment the components were mixed. Other tests, namely thermal analysis and FTIR spectroscopy, were performed after 24 h of hydration.

The cement paste was prepared as follows: cement and distilled water (water-to-cement ratio = 0.5) were mixed by hand. The cement slurry was then poured into a small plastic bag and sealed. After 24 h of hydration at room temperature, the paste was removed from the bag and subjected to further processing.

### 2.4. Methods, Devices, and Measurement Conditions

* Calorimetry measurements were performed using Calmetrix I-Cal 2000 HPC (Calmetrix, Boston, MA, USA). This is a two-channel isothermal calorimeter that measures the heat of hydration of a given material. A temperature of 23 °C was used for stabilization and baseline measurement. Gypsum pastes and cement paste were placed in calorimeter 1 min after adding water to the binder.
* Thermal analysis (TG/DTG/DTA) was conducted using STA 2500 Regulus (Netzsch, Selb, Germany) thermoanalyzer, measurement temperature range: 30–800 °C, the rate of heating: 10 °C min^−1^, gas atmosphere: nitrogen, open alumina crucible, the mass of the sample: 15–20 mg. Before measurement, hydration was inhibited using acetone. Then the samples were dried. Free water was removed in this way.
* Infrared spectroscopy (FTIR)—spectra were collected using Genesis II (Mattson, Madison, WI, USA) spectrometer at wavenumbers range: 4000–400 cm^−1^. Samples for testing were prepared in the same way as for thermal analysis tests. Then KBr pellets were prepared.
* Compressive and bending strength tests were carried out for gypsum samples formed into bars of the size 4 × 4 × 16 cm. Strength tests were performed after 28 days on a modernized press Hackert ZD10/90 (Fritz HECKERT, Chemnitz, Germany). Before the tests, the samples were stored at 20 ± 1 °C.

## 3. Results and Discussion

### 3.1. Calorimetric Measurements

**Figure 2:**
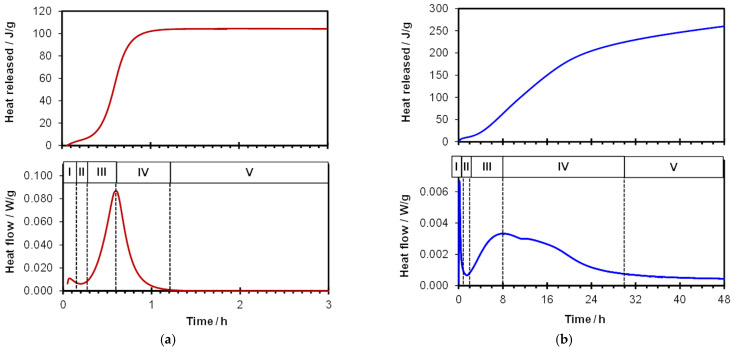
Exemplary results of calorimetric tests for gypsum (**a**) and Portland cement (**b**) (I–V—explanations in the text).

The setting and hardening processes of gypsum and cement are based on reactions with water. The hydration of both binders is exothermic [2,4,8], meaning that heat is released immediately upon the addition of water, which is confirmed by the results obtained in this work. Figure 2 shows a comparison of typical calorimetric curves and the cumulative heat released for gypsum and cement. The shapes of the heat release curves are typical for such binders. Several characteristic stages can be distinguished [2,3,4,5,8,17,18,19,20,21,22]:

I—The wetting period, during which wetting of the grains and dissolution of some components take place. In the case of the gypsum binder, rapid dissolution of part of the bassanite occurs. The solution becomes saturated with respect to Ca^2+^ and SO_4_^2−^ ions, and the first portions of the hydrated product may precipitate. Similarly, in Portland cement paste, the solution becomes saturated with Ca^2+^ and SO_4_^2−^ ions (although the sulfate concentration in the pore solution of cement paste is lower than in gypsum paste), hydration processes begin, and ettringite forms.
II—Induction period: Heat release is very low; nuclei of hydrates are formed.
III—Acceleration period, during which significant heat release occurs (more intense in the case of gypsum compared with cement). Precipitation of products takes place: in gypsum paste, CaSO_4_·2H_2_O, and in cement paste, the C–S–H phase and Ca(OH)_2_. The beginning of this period marks the onset of setting, and before the maximum peak is reached, final setting occurs.
IV—Deceleration period: Hydration processes occur predominantly in the solid phase, making them less intense.
V—Stabilization period: Hydration products continue to crystallize and grow, forming a hardened structure, while unhydrated particles gradually disappear.

The periods mentioned above are marked on the heat-flow curves for gypsum and cement in Figure 2. As can be seen from the results, the heat-release curves for both materials follow similar courses and can be divided into comparable periods. However, a significant difference concerns the time required for setting and for the formation of a hardened structure. In the case of gypsum, the exothermic heat-release process occurs much faster than in cement, and after 12 h no further changes in the shapes of the recorded curves are observed (which is why Figure 2a,b use different horizontal-axis scales). Thus, gypsum sets much faster than cement.

**Figure 3:**
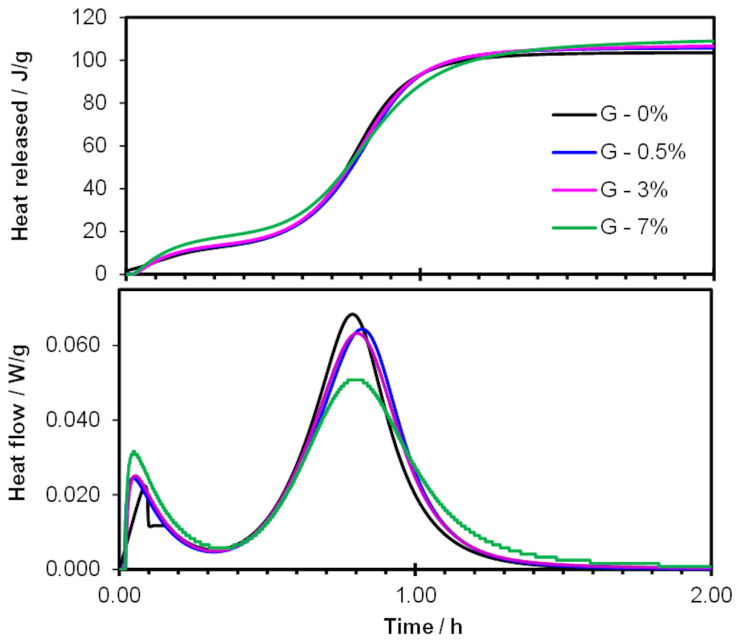
Curves of heat release rate and cumulative heat released for gypsum containing 0, 0.5, 3 and 7% of HEMC.

Gypsum setting depends on the kinetics of bassanite dissolution and the subsequent precipitation of the dihydrate, i.e., gypsum. These processes can be disrupted in the presence of cellulose ethers, as indicated in [23]. The authors of [23] suggest that the dissolution mechanism is controlled by diffusion in both distilled water and cellulose ether solutions. They also note that the admixture delays bassanite dissolution and prolongs the gypsum nucleation time. This effect can be explained by various mechanisms of action associated with cellulose ethers [17]. They increase the viscosity of the paste and may adsorb onto unhydrated grains, thereby impeding the movement of Ca^2+^ and SO_4_^2−^ ions, which in turn prolongs bassanite hydration [17]. As shown in this study, the introduction of HEMC into the system alters the kinetics of heat release (Figure 3), thus influencing the early hydration process. The intensity of the main heat-release effect associated with the crystallization of dihydrate gypsum decreases with increasing amounts of the introduced polymer. Furthermore, the peak maximum shifts toward slightly longer hydration times. There are no significant differences in the end time of the induction period, indicating that the modified samples and the reference paste begin to set at a similar time. However, the slope of the curves in the acceleration period (III) decreases with increasing admixture content, which, together with the shift in the peak maximum, indicates a delay in hydration and setting. Analysis of the deceleration period (IV) shows that the heat-release rate decreases more rapidly for the reference sample than for the mixtures containing HEMC, thus the crystallization process is extended in the presence of HEMC. The phenomenon of slowed formation of gypsum crystal structures in the presence of cellulose derivatives, compared to samples without admixtures, has also been reported by other authors [3,9].

The total heat released during the studied period is similar for all samples. However, a slight upward trend with increasing HEMC content can be observed. The highest admixture dosage (7%) leads to an increase in heat release already in the first minutes of hydration. The results obtained in this study confirm the general relationships observed in our previous work [11,12,24]. Certain differences (e.g., the duration of the induction period in the presence of HEMC) may stem from variations in measurement conditions, the amount of HEMC introduced, and the equipment used.

HEMC, like other cellulose derivatives [3], can absorb free water, as mentioned earlier. As a result, a reduction in the effective water/gypsum ratio can be expected, hindering the dissolution of the hemihydrate and the subsequent formation of gypsum dihydrate. However, with lower water content, the solution becomes supersaturated more quickly, and gypsum crystal nuclei can form more readily. Thus, hydration may ultimately be accelerated, as Zhang et al. point out for HPMC [8]. In this study, a high water/gypsum ratio of 0.75 was used, which exceeds the theoretical requirement. This likely explains why the effect of HEMC on early hydration was not significant. Moreover, instead of an accelerating influence, a retarding and hindering effect on gypsum hydration was observed, as indicated by the shift in the maximum of the main peak on the calorimetric curve toward longer hydration times. This effect is insignificant at admixture amounts up to 3% and becomes more pronounced at higher HEMC content (7%). Another mechanism delaying bassanite hydration may be polymer adsorption onto binder grains, which limits water access [7]. Therefore, the overall effect of HEMC on early gypsum hydration and setting results from the combined influence of these various interactions.

### 3.2. Thermal Analysis

**Figure 4:**
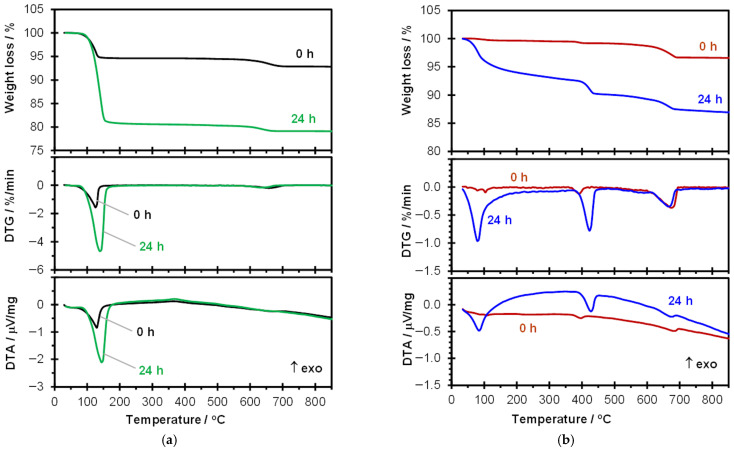
Exemplary results of TG, DTG and DTA curves for raw materials: bassanite (**a**) and Portland cement (**b**) and after 24 h hydration.

Figure 4 shows a comparison of the TG, DTG and DTA curves obtained for raw materials, i.e., bassanite (Figure 4a) and cement (Figure 4b), and after hydration for 24 h.

On the thermogravimetric curve of bassanite, two endothermic weight-loss effects can be distinguished: an approximately 6% loss in the temperature range of 80–140 °C and a smaller one in the range of 550–700 °C. The first effect is related to the dehydration of bassanite, while the second results from the decomposition of CaCO_3_ and indicates a small amount of this contaminant in the sample. The exothermic peak at around 365 °C, not associated with any mass change, results from the structural transformation of anhydrous CaSO_4_ [25]. This corresponds to the phase transformation from γ-CaSO_4_ to β-CaSO_4_ (these sulfates are also referred to as CaSO_4_ III and CaSO_4_ II, or soluble and insoluble CaSO_4_, respectively) [26]. The TG, DTG, and DTA curves are typical for bassanite and consistent with those previously published by other authors [25].

Portland cement contains only a few percent of gypsum; therefore, only a small mass loss is visible on the TG curve in the temperature range up to 150 °C, which is confirmed by a minor effect on the DTG curve. Another small endothermic mass loss at around 400 °C confirms the presence of Ca(OH)_2_ and results from its dehydroxylation. Similarly to the bassanite sample, the cement also exhibits a mass loss at 600–700 °C due to the decomposition of carbonates.

After 24 h of contact with water, the thermal curves for both materials change, which is a typical effect resulting from hydration processes. In the case of bassanite exposed to water for 24 h and subsequently subjected to free-water removal, two endothermic effects can also be observed on the TG curves, similar to the raw material. However, the mass loss occurring at lower temperatures (80–180 °C) is approximately three times greater than that of the hemihydrate. This results from the prior hydration of the hemihydrate and the formation of gypsum dihydrate. The observed mass loss is therefore associated with gypsum dehydration. This dehydration process is sensitive to the test conditions, especially water vapor pressure [25,27]. Consequently, different thermal analysis results appear in the literature depending on the measurement conditions: two-stage dehydration (separate or partially overlapping effects on the TG and DTA curves, e.g., in [17,25,27,28]) or a single mass loss accompanied by one endothermic effect on the DTA curve, as in this study and in some reports by other authors [25,26,27]. The exothermic effect associated with CaSO_4_ conversion is visible in both the raw material and the hydrated gypsum.

Portland cement after 24 h of hydration shows the presence of hydration products, as evidenced by endothermic mass losses in the temperature range up to 200 °C (dehydration of ettringite and the C–S–H phase) and in the range of 380–450 °C (dehydroxylation of Ca(OH)_2_).

**Figure 5:**
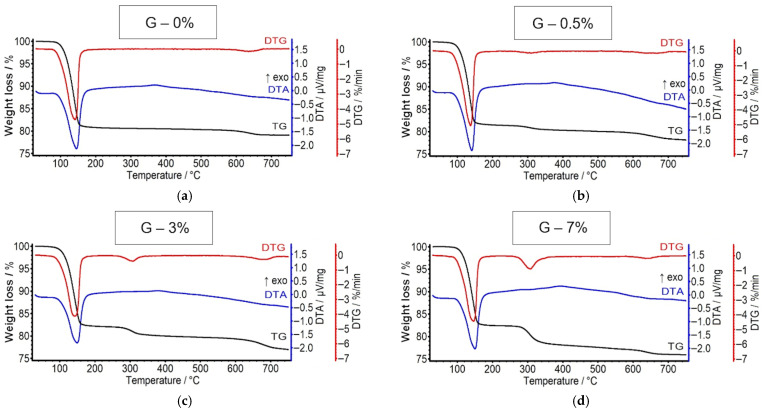
TG, DTG and DTA curves for hydrated gypsum (24 h) containing 0% (**a**), 0.5% (**b**), 3% (**c**) and 7% (**d**) of HEMC.

Figure 5 presents the TG, DTG, and DTA curves for the gypsum binder modified with HEMC. It can be seen that the recorded curves are similar for all samples. The additional mass loss at approximately 300 °C in the modified gypsums results from the presence of the polymer. Apart from this effect, no additional signals are observed that would indicate the formation of other phases in the presence of the admixture. According to [7], HPMC may alter the structure of the hardened material, but it does not become incorporated into the crystalline structure of gypsum. A similar effect can therefore be expected for HEMC.

### 3.3. Infrared Spectroscopy

**Figure 6:**
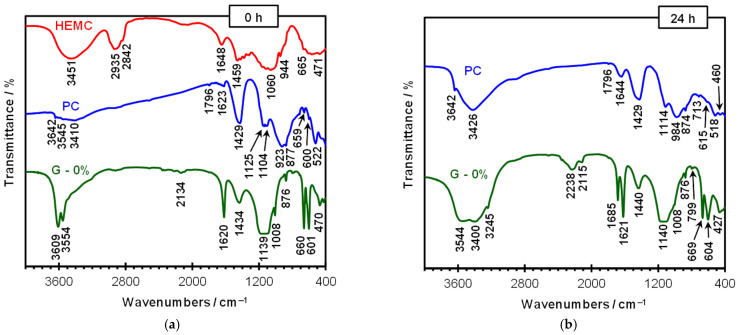
Comparison of FTIR spectra of Portland cement (PC), bassanite/gypsum (G-0%), and HEMC: (**a**) raw materials; (**b**) hydrated gypsum and Portland cement (24 h after adding water).

FTIR spectra of the materials used—namely Portland cement, dry gypsum (specifically bassanite, CaSO_4_·0.5H_2_O), and HEMC—are presented in Figure 6. For comparison, Figure 6 also shows the FTIR spectra of hydrated gypsum and Portland cement 24 h after the addition of water.

A few bands typical of CaSO_4_·0.5H_2_O are visible in Figure 6. Two intense, sharp bands at approximately 3610 and 3555 cm^−1^ correspond to O–H stretching vibrations (H–O–H). Another band confirming the presence of water molecules in the sulfate structure appears at 1620 cm^−1^ (O–H bending). The main sulfate band occurs in the wavenumber range 1050–1250 cm^−1^, with a maximum at around 1140 cm^−1^. It corresponds to the antisymmetric stretching vibrations of the SO_4_ groups. Additional bands characteristic of the tested material include three bands of antisymmetric bending vibrations at 660, 629, and 601 cm^−1^ (the band at 629 cm^−1^ overlaps with the one at 601 cm^−1^ and therefore does not appear as a separate peak, forming only a small shoulder on the latter), as well as weak bands in the range 2000–2500 cm^−1^ (overtones) [29]. Moreover, bands indicating the presence of calcium carbonate are observed: a broad, medium-intensity band at approximately 1435 cm^−1^ (asymmetric stretching vibrations of carbonate groups) and a sharp one at about 875 cm^−1^ (bending vibrations) [29], which is consistent with the results of the thermal analysis.

The presence of small amounts of calcium sulfate in cement is also evident in the IR spectrum. The strong, overlapping peaks at 1140, 1125, and 1104 cm^−1^ indicate stretching vibrations of SO_4_ groups in gypsum and bassanite [29]. The bands at approximately 600 and 660 cm^−1^ further confirm the presence of bassanite. The IR spectrum of cement also shows bands typical of calcium carbonate: an intense band at 1429 cm^−1^, a sharp band at 714 cm^−1^, and a weak one at 1796 cm^−1^. Other bands are associated with silicates (alite, belite), which constitute the main components of cement. Specifically, a broad, intense band in the wavenumber range 750–1000 cm^−1^, with maxima at approximately 923 and 877 cm^−1^, results from stretching vibrations of SiO_4_^4−^ tetrahedral units, and the band at about 525 cm^−1^ corresponds to bending vibrations of O–Si–O bonds [29]. In addition, a very weak band at 3642 cm^−1^ (OH stretching) confirms the presence of small amounts of Ca(OH)_2_ in the cement.

HEMC is an organic compound; therefore, its IR spectrum differs completely from the spectra of bassanite and cement discussed above. Its chemical formula is shown in Figure 1. In fact, the only similar bands are those associated with stretching and deformation vibrations of water molecules: a broad band with maxima at approximately 3450 cm^−1^ (also encompassing O–H vibrations within the structure of the compound) and at 1650 cm^−1^. The overlapping bands at about 2845 and 2935 cm^−1^ correspond to stretching vibrations of C–H bonds in CH_3_ and CH_2_ groups. A series of bands in the wavenumber range 1300–1460 cm^−1^ corresponds to deformation vibrations of these groups. Another group of intense bands in the range 900–1300 cm^−1^ arises from stretching vibrations of C–O–C bonds. In this wavenumber range, around 1200 cm^−1^, the presence of C–O–CH_3_ deformation vibration band can also be expected [3]. The FTIR spectrum for HEMC confirms the presence of ether and alkyl groups in the molecular structure.

Comparing the IR spectra of the materials used in this study in the range of bands common to all of them—namely the O–H vibration bands in H–O–H—it can be observed that the differences in the deformation vibration band are insignificant, whereas clear changes are visible in the stretching vibration band. This indicates a different mode of water binding in the samples and a different extent of hydrogen bonding.

**Figure 7:**
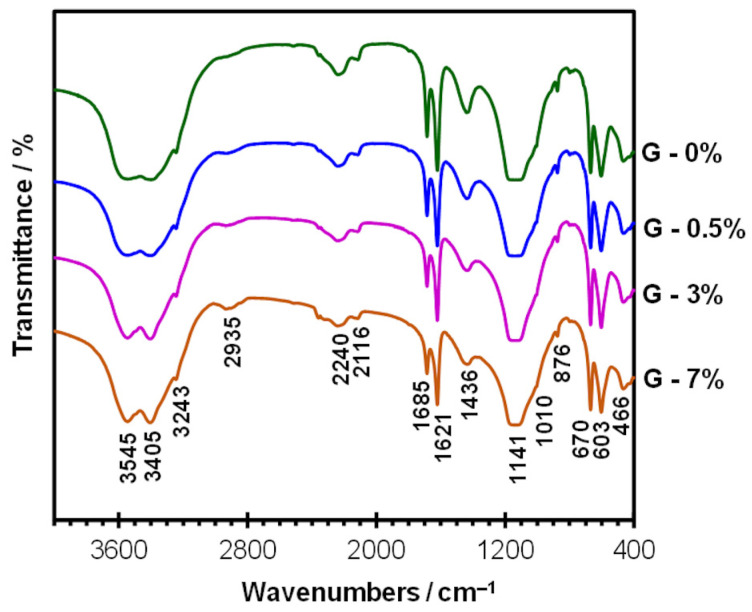
FTIR spectra for hydrated gypsum (24 h) containing 0, 0.5, 3 and 7% of HEMC.

After the hydration of bassanite, several absorption bands change due to the chemical processes taking place and the formation of gypsum dihydrate (Figure 6 and Figure 7). It can be clearly seen that the H–O–H stretching vibration band becomes broader, and the peak positions shift to approximately 3545 and 3400 cm^−1^, the latter indicates gypsum. The O–H bending vibrations appear as two bands (unlike in the hemihydrate): at about 1685 and 1620 cm^−1^. The main band exhibits a maximum at approximately 1140 cm^−1^. Two bands characteristic of gypsum dihydrate are clearly visible at 669 and 604 cm^−1^ [3,29]. The overtone bands are more intense compared with the hemihydrate spectrum, and peaks can be distinguished at approximately 2240 and 2115 cm^−1^.

For the Portland cement paste (Figure 6), the IR spectrum recorded after 24 h of hydration is completely different from that of gypsum, as expected. The IR spectrum of the cement paste no longer shows the presence of calcium sulfate, which reacted with aluminate and calcium ions to form ettringite, as indicated by a strong band at approximately 1115 cm^−1^. Changes in the shape of the silicate band—specifically, the separation of the band with a maximum at about 980 cm^−1^—indicate the formation of the C–S–H phase. A broad band with a maximum at approximately 3425 cm^−1^ corresponds to the presence of water bound in hydrates. Furthermore, the IR spectrum of the cement paste shows bands confirming the presence of CaCO_3_ as well as Ca(OH)_2_ formed during hydration.

Figure 7 shows the results for hydrated gypsum containing the polymer admixture. The spectra recorded for the HEMC-modified samples are similar. The presence of the polymer is confirmed by bands at 2900–2935 cm^−1^ (C–H vibrations), which are not visible in the spectrum of the unmodified sample. The observed changes in the shape of the H–O–H stretching vibration bands, which become more pronounced with higher polymer content, indicate the influence of HEMC on water binding within the structure. According to [3], the hydrophilic groups in the HEMC structure act as traps for water molecules, thereby retaining them.

### 3.4. Mechanical Strength Tests

**Figure 8:**
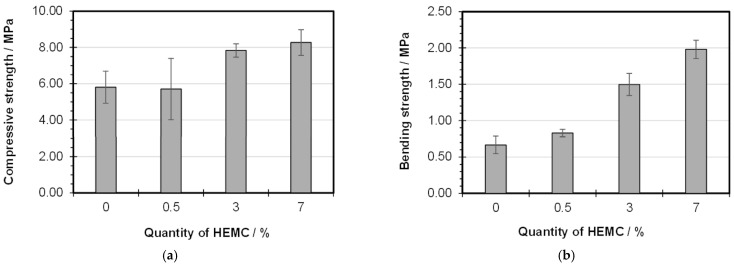
Compressive (**a**) and bending (**b**) strength of HEMC-modified gypsum pastes.

Figure 8 shows the effect of the HEMC on the mechanical properties of gypsum pastes. The mechanical properties of hardened gypsum are influenced by reaction kinetics, including the rate of bassanite dissolution and product precipitation. These parameters, in turn, affect the microstructure of the final material. This study confirmed the retarding effect of HEMC; therefore, one would expect a deterioration in mechanical properties, as observed in previous studies [28]. However, the results shown in Figure 8 indicate that the presence of the admixture improves the strength of the final material. The introduction of 0.5% HEMC into the system results in minimal changes in compressive strength compared with the reference sample, but leads to an increase in bending strength. Higher amounts of admixture (3% and 7%) result in an increase in both bending and compressive strength. The increase in bending strength suggests that the presence of the admixture enhances resistance to scratches caused by material shrinkage.

The authors of [5] demonstrated that the bending stress increases with increasing HEMC content in the gypsum paste. The improvement in mechanical properties may result from changes in the microstructure as well as hydrogen bonds which can be formed between the appropriate groups of polymer and surface of gypsum [7]. Zhi et al. [17] observed that adding cellulose ethers to gypsum initially leads to an increase in 1-day compressive strength, followed, beyond a certain dosage, by a decrease. The mechanical properties of gypsum with admixture may be reduced not only due to the delayed hydration of bassanite but also due to the formation of a hardened structure with an increased number of air bubbles, which increases the porosity of the material [17].

The mechanism of action of the polymer admixture, including water retention, can in some cases beneficially modify the properties of the final material. A polymer film is probably formed in the modified gypsum system [5]. As mentioned earlier, the HEMC admixture in gypsum mortar retains water and prevents rapid drying of the material, so reduced susceptibility to fine cracking can be expected. Furthermore, the polymer particles exist in the form of three-dimensional chains, which can reduce shrinkage stress; therefore, cracking can also be reduced. It can be expected that the cellulose ether chains distributed throughout the gypsum structure may improve its elasticity and bending strength.

The results obtained in this work, compared with other literature reports, indicate once again that the interaction of the polymer within the hydrating gypsum system depends on various factors, including the amount of admixture, its molecular weight, the water-to-gypsum ratio, the type of gypsum (bassanite) used, and its properties such as grain size and specific surface area, etc. Under the experimental conditions used in this study, the retardation effect observed in the presence of HEMC is not significant, and therefore did not negatively affect the compressive strength. It was also noted that the HEMC admixture extends the crystallization time of gypsum, which undoubtedly influences its microstructure. It is possible that slower crystal growth in the presence of HEMC may lead to the precipitation of smaller crystals with a more uniform distribution and better packing, as well as fewer shrinkage microcracks, resulting in higher strength of the final gypsum product. The formation of smaller gypsum crystals and the improvement of crystal agglomeration in the presence of HEMC, compared with the hydration products of hemihydrate without admixture, were reported by other authors [3,5,9]. This issue requires more detailed analysis in future research.

## 4. Conclusions

Understanding the mechanisms of bassanite hydration in the presence of polymer admixtures has not only scientific, but also practical implications, enabling the prediction of material properties and their adaptation to specific applications. Analysis of the literature reports and the results of the research conducted in this work showed that the effect of the HEMC admixture on the properties of gypsum materials may vary depending on various factors, including the amount of admixture and its characteristics. The following conclusions can be drawn from the results and analyses obtained in this study:

1. The heat-release curves of gypsum hydration and gypsum hydration in the presence of HEMC show a shape typical of this type of binder. The addition of HEMC results in delayed hydration and prolonged crystallization of gypsum. This effect is particularly noticeable at higher admixture contents. The total heat released increases slightly with increasing HEMC content.
2. The results of thermal analysis and infrared spectroscopy do not indicate the formation of any new phases in the gypsum paste containing the admixture. The presence of the polymer admixture in the system is visible in both the thermal analysis and FTIR results. HEMC probably does not incorporate into the crystalline structure of gypsum, but it causes changes in the microstructure and leads to differences in the mode of water binding and in the extent of hydrogen bonding.
3. The presence of HEMC can improve the bending and compressive strength of the final material. This is likely due to slower precipitation of gypsum crystals, which are smaller in size and result in a denser structure. Polymer chains also contribute to improved bending strength. However, since the results reported by different authors are contradictory, this issue requires more detailed consideration in future studies.

## Data Availability

The original contributions presented in this study are included in the article. Further inquiries can be directed to the corresponding author.
